# Photoperiod-driven rhythms reveal multi-decadal stability of phytoplankton communities in a highly fluctuating coastal environment

**DOI:** 10.1038/s41598-022-07009-6

**Published:** 2022-03-10

**Authors:** Lorenzo Longobardi, Laurent Dubroca, Francesca Margiotta, Diana Sarno, Adriana Zingone

**Affiliations:** 1 Integrative Marine Ecology Department, Stazione Zoologica Anton Dohrn, Villa Comunale, 80121 Naples, Italy; 2 Institut Français de Recherche Pour l’Exploitation de la Mer, IFREMER, Laboratoire Ressources Halieutiques de Port-en-Bessin, 14520 Port-en-Bessin-Huppain, France; 3 Research Infrastructures for Marine Biological Resources Department, Stazione Zoologica Anton Dohrn, Villa Comunale, 80121 Naples, Italy

**Keywords:** Ecology, Environmental sciences, Ecology, Community ecology, Ecological modelling, Ecosystem ecology, Ocean sciences, Marine biology

## Abstract

Phytoplankton play a pivotal role in global biogeochemical and trophic processes and provide essential ecosystem services. However, there is still no broad consensus on how and to what extent their community composition responds to environmental variability. Here, high-frequency oceanographic and biological data collected over more than 25 years in a coastal Mediterranean site are used to shed light on the temporal patterns of phytoplankton species and assemblages in their environmental context. Because of the proximity to the coast and due to large-scale variations, environmental conditions showed variability on the short and long-term scales. Nonetheless, an impressive regularity characterised the annual occurrence of phytoplankton species and their assemblages, which translated into their remarkable stability over decades. Photoperiod was the dominant factor related to community turnover and replacement, which points at a possible endogenous regulation of biological processes associated with species-specific phenological patterns, in analogy with terrestrial plants. These results highlight the considerable stability and resistance of phytoplankton communities in response to different environmental pressures, which contrast the view of these organisms as passively undergoing changes that occur at different temporal scales in their habitat, and show how, under certain conditions, biological processes may prevail over environmental forcing.

## Introduction

Given the tremendous importance of phytoplankton in the functioning and health of the oceans, one of the challenges of the modern scientific community is to understand and quantify their responses to a constantly changing environment. The impact of physical and chemical forcing on biological communities has been changing rapidly in the last decades^1^ and is also predicted to become more severe in the next years due to the human-induced increase in global average temperature^2^. Changes in physical and chemical factors in the marine environment can affect phytoplankton dynamics acting directly on species physiology^3^ and by changing the physical habitat of the autotroph communities^4^, resulting in modifications of light and nutrient availability by which phytoplankton is ultimately governed.

Understanding the mechanisms that underlie the relationships between the environment and phytoplankton is particularly important in coastal areas. Land-sea interface areas represent one of the most productive types of environment and are among the most ecologically and socio-economically important systems on the planet^5^. Half of the world's population lives along the coasts^6^, whereby human well-being is directly linked to phytoplankton through sea-food availability, overall environmental quality and ecosystem services that coastal environments provide^7^. At the same time, the pace of change in coastal-estuarine areas is accelerating^8^, while the growth of the human population is increasing the anthropic impact and the need to exploit marine resources.

The influence of land in coastal systems translates into very complex ecological dynamics, which are hardly understandable and scarcely predictable^9^. The relationship between environmental fluctuations and phytoplankton has been addressed in terms of variability in the seasonal cycle^10^, responses to pulse events^11^ and long-term changes^12–15^. Given the remarkable diversity of coastal environments^7^, different factors may have different importance among places in shaping the phytoplankton community. Nutrient load and temperature fluctuations in the Baltic Sea are considered the main factors altering the temporal structure of the community^16,17^, whereas turbidity can affect community composition by modifying the light environment in other shallow systems^15,18,19^. Long-term observations in Ilha Grande Bay (Brazil) show that the microalgal annual cycle and composition are markedly shaped by atmospheric conditions, such as rain and wind patterns^20^. In other cases, no strong links have been found between important interannual changes in environmental conditions and changes in the phytoplankton community^21^, suggesting that other factors such as trophic processes may play a more important role. Collectively, these studies have highlighted an impressive complexity of ecological dynamics related to the multidriver nature of environmental change and, not least, the technical challenges and limitations faced by the acquisition and comparability of long-term high-quality phytoplankton data^22^.

Despite the high degree of variability in the coastal habitat, in many cases temporal regularity has also been reported for phytoplankton across many levels of biological organization, encompassing species successional patterns and associations^23^, functional groups and species traits^24,25^ and massive events such as blooms^26^. A seasonal signal in the occurrence of marine autotrophs is typical of mid and high-latitude systems, where the annual astronomical cycle of solar radiation and day length triggers and shapes the times for several biological dynamics^9,27^ At the base of the marine food web, temporal phytoplankton patterns set the pace for the dynamics of high-trophic levels and biogeochemical processes^28^, whereby their modifications are closely coupled with the stability and the general ecological processes in marine systems^29^.

Seasonal and interannual patterns of phytoplankton variability have usually been investigated based on bulk and aggregated indicators such as chlorophyll *a* and functional groups, which are useful to identify changes over wide spatial and temporal scales^30^ but can provide a solid interpretative framework of temporal phytoplankton changes only if integrated with taxonomic data^31^. In trait-based approaches, the close relationships between environmental fluctuations and distribution of species traits across the seasons reflect the match of species’ physiological settings with environmental conditions^32,33^. However, due to the scarcity of marine long-term programs^34^ and also to a growing scarcity of taxonomic experts, a gap exists in the knowledge of the phenological and interannual dynamics of phytoplankton species and communities and their interactions with the environmental variability.

The Gulf of Naples represents a suitable site for the study of seasonal and interannual variations of phytoplankton species and assemblages, because of the availability of one of the longest, high-resolution time series of data which is collected at the Long Term Ecological Research site MareChiara (LTER-MC) since 1984. In a global comparison of planktonic systems based on chlorophyll data, the site has been shown to be highly variable at both the seasonal and interannual scale^9^, which is compatible with its nature of a mid-latitude coastal site not far from one of the most populated areas of the Mediterranean Sea^35^. In the Gulf of Naples, temporal species recurrence against environmental variability was noticed for several phytoplankton and zooplankton species and further investigated for copepod populations^36^. Here, taking advantage of the high resolution of the taxonomic data, we extend the search for temporal occurrence patterns to a high number of phytoplankton species and their assemblages. Specifically, using the whole time series from 1984 to 2015, we (i) characterize the seasonal patterns and trends of the main environmental variables and bulk phytoplankton features (chlorophyll *a* and total cell numbers), (ii) analyse the occurrence patterns of individual phytoplankton species and their communities, (iii) characterise the coupling between community turnover and environmental variability, and (iv) provide a hierarchical assessment of the environmental factors involved in the temporal phytoplankton variability. Our aim is to verify, in a highly dynamic and variable coastal environment, whether phytoplankton species and communities are also variable and unpredictable or, rather, they show recurrent and stable phenological patterns over the years, which would indicate the relevance of biological processes linked to species-specific life-cycle traits in determining their seasonal succession.

## Results

### Seasonal variability and trends in environmental variables, chlorophyll a and phytoplankton abundance

**Figure 1:**
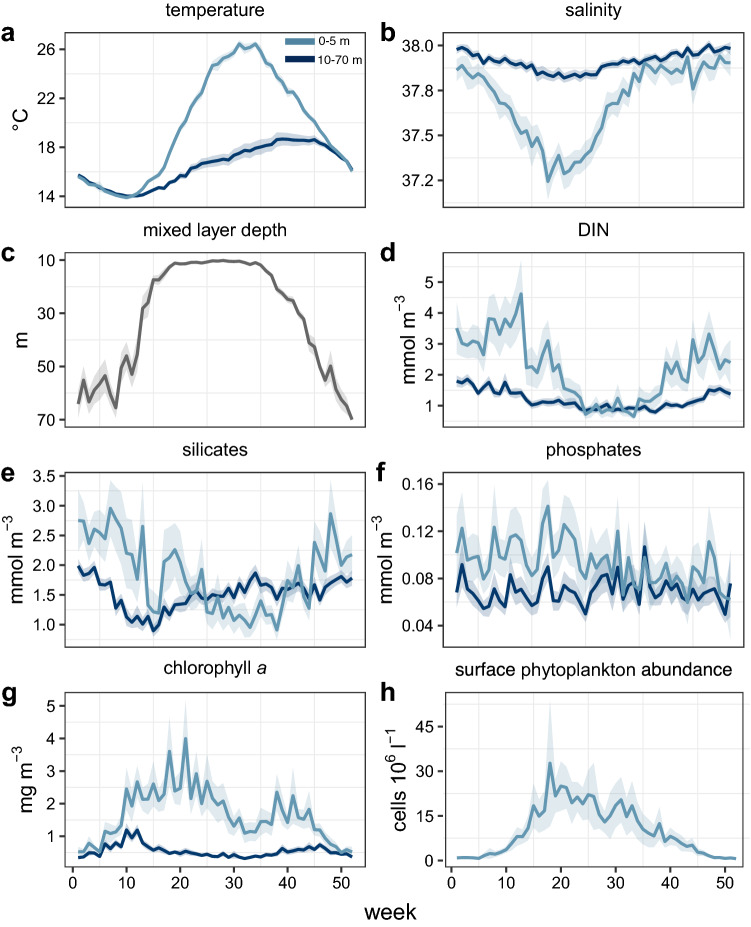
Weekly climatology of the main environmental and biological variables at LTER-MC during 1984–2015. In all panels with two lines, the light and dark blue lines refer to the average of surface (0–5 m) and deep layer (10–70 m) respectively, like in panel a, while the shaded areas represent the respective 0.95 confidence interval.

A strong seasonal signal is a main characteristic of the system under study, where surface (0–5 m) water temperature was characterized by winter minima (> 13.13 ± 0.58 °C) and mid-late summer maxima (< 27.79 ± 0.76 °C) (Fig. 1a). During winter, the water column was homothermic, whereas from mid-spring to early autumn surface layer temperatures were significantly higher than those recorded below 10 m. Salinity (Fig. 1b) showed wide seasonal fluctuations with the maxima (up to 38.31) generally in early autumn, when high seawater temperature and enhanced winds concurred to increase the evaporation rate. The lowest salinity values (35.47–36.20) were typical of late spring and early summer. Below 10 m depth, salinity followed the same pattern as at surface but with an overall lower variability around the mean compared to the surface. The mixed layer depth (Fig. 1c), mainly driven by temperature, shallowed quickly between mid-March to the end of May and settled around the 10-m depth until the beginning of September, after which it gradually deepened occupying the entire water column in December.

Except for phosphates (Fig. 1f), nutrient concentrations in the water column showed wide seasonal variations, with the highest DIN (Fig. 1d) and silicate concentrations (Fig. 1e) recorded in winter and followed by a gradual decline towards the summer minima. In the deep layers (10–70 m), nutrient concentrations were typically lower and less variable than in surface layers except for summer, when DIN was low throughout the whole water column and silicates were higher in deep layers than at the surface.

Since mid-late winter, surface chlorophyll *a* concentrations increased gradually reaching their maximum values generally in May, then decreased in summer (Fig. 1g). Seasonal variations were smoother in deeper layers, which were not affected by the late spring–summer peaks. The break of the thermocline in late summer-early autumn (Fig. 1c) was generally accompanied by a second chlorophyll *a* increase of lesser magnitude than in spring. Surface phytoplankton abundance started to increase in late winter and raised rapidly to reach the annual maximum (up to 10^7^–10^8^ cells L^−1^) in late spring, usually in May (Fig. 1h). It kept relatively high throughout the summer and gradually dropped to the lowest values (down to the minimum 10^4^ cells L^−1^) in late autumn.

Although seasonal cycles were well defined for most abiotic and biotic variables, the relatively wide confidence interval around weekly averages revealed a high interannual variability, especially throughout the stratification period, also concerning total phytoplankton abundance and chlorophyll *a*. Phytoplankton communities were numerically dominated by small phytoflagellates and small diatoms in almost all seasons (Supplementary Fig. S2), while dinoflagellates reached the highest densities between April and May and coccolithophores increased in late summer, generally reaching their maximum in October.

**Figure 2:**
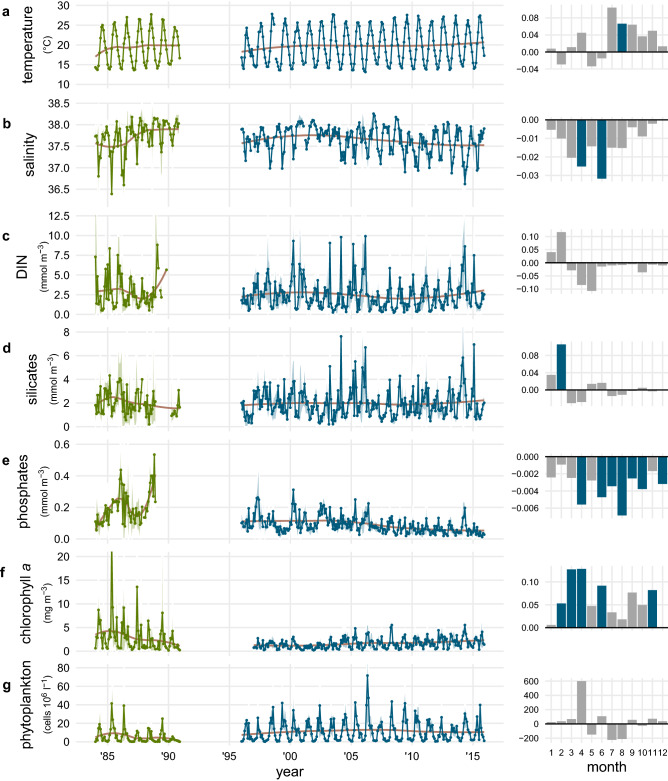
(Left) Time series of monthly averaged values of environmental and phytoplankton data at LTER-MC. The red line is the local polynomial regressions fitted to each time series while the shaded area around means represents the 0.95 confidence interval. (**a**) temperature, (**b**) salinity, (**c**) DIN, (**d**) silicates, (**e**) phosphates, (**f**) chlorophyll *a*, (**g**) phytoplankton abundance. (Right) Average annual change of each variable at monthly scale during the continuous sampling period 1996–2015. The significance of linear monotonic trends (Mann–Kendall test) is indicated by the colours of the bars, where grey bars refer to a pvalue higher than 0.05 while blue bars are significant at pvalue < 0.05.

Concerning long-term variations, a surface temperature increase at LTER-MC was persistent and particularly evident in the second half of the year (Fig. 2a), characterised by a significant linear upward trend in August (average >  + 0.06 °C year^−1^) and by a consistent increase also in July and September (+0.01and +0.06 °C year^−1^, respectively). Conversely, surface salinity reached the minima in the first years of the time series and decreased in all months over the second part (Fig. 2b), showing a significant linear downward trend in April and June, and interannual, cyclical fluctuations characterised by a period of about 5 years. Surface DIN and silicates (Fig. 2c–d) were slightly higher in the first part of the series and remained quite stable over the years, except for a significant linear increase of silicates in February and an overall decrease of DIN during late winter. Phosphate concentrations were much higher in the first part of the series and underwent a gradual but remarkable decline since the ‘90s, which was significant in all seasons but winter (Fig. 2e). In parallel with the main nutrients, surface chlorophyll *a* concentration showed the highest peaks during the first part of the series (Fig. 2f). On average, the period 1997–2003 was characterized by the lowest phytoplankton biomass, followed by a gradual increase resulting in a linear and positive trend in many periods of the year. Total phytoplankton abundance was generally lower during the first part of the series (Fig. 2g) despite high values of chlorophyll *a*. The analysis of the monthly abundance trends showed a generally stable pattern, except for an increase in April and a slight decrease in the summer months, namely in July and August.

### Temporal regularity in phytoplankton species and communities

The randomization test performed on the results of the Lomb-Scargle periodogram analysis identified 75 species as significantly periodic out of a total of 81 species investigated (Supplementary Table S1). The temporal variability in the turnover of those taxa was investigated using the Bray–Curtis autocorrelation analysis, in which we estimated the communities’ similarity between pairs of samples for each possible monthly time-lag along the time series (1–384 months over the 32 years). The analysis performed on the second part of the time series (1996–2015, Fig. 3) as well as on the whole time-series (Supplementary Fig. S3) highlighted a clear seasonal rhythm in community composition, which showed a maximum average similarity among communities sampled at a time distance of 12 months and their multiples (24, 36, and so on), and minima at 6, 18 and 30 months apart. Such recurrent pattern in the similarity of the community was kept along all the length of series, with a slight decay in both minimum and maximum values along the years over the 1996–2015 period, possibly related to the decrease of data available for the comparison between communities far in time by more and more years. When the first years (1984–1991) were included in the analysis, the similarity decay became a significant downward monotonic trend (pvalue < 0.001), with lower maximum and minimum average similarity values (Supplementary Fig. S3). Along with its difference from the one obtained on the years 1996–2015, this result reflected the higher difference of phytoplankton communities of the first years from the rest of the time series.

**Figure 3:**
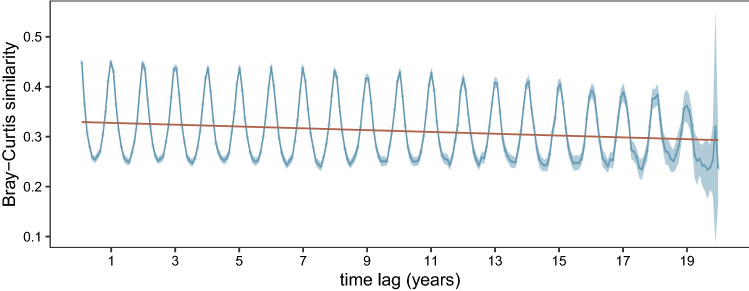
Interannual recurrence of phytoplankton communities shown by average. Bray–Curtis similarities (0: completely different communities; 1: identical communities; shaded area: 95% confidence interval) between all pairs of weekly samples separated by a given number of months of the series (time lag, x axis) during 1996–2015. The first point of the series represents the average and 95% confidence interval of the Bray–Curtis similarity values among communities sampled 30 ± 4 days apart in the time interval considered. Similarly, the second point represents the average Bray–Curtis similarity among all the communities sampled 60 ± 4 days apart, the point at 1 year is the average similarity among all the communities sampled at 360 ± 4 days apart, and so on. The decay of maximum and minimum average similarity values over time is probably influenced by the lower number of samples to compare for time lags of many years.

### Comparing environmental and phytoplankton community variability across years

**Figure 4:**
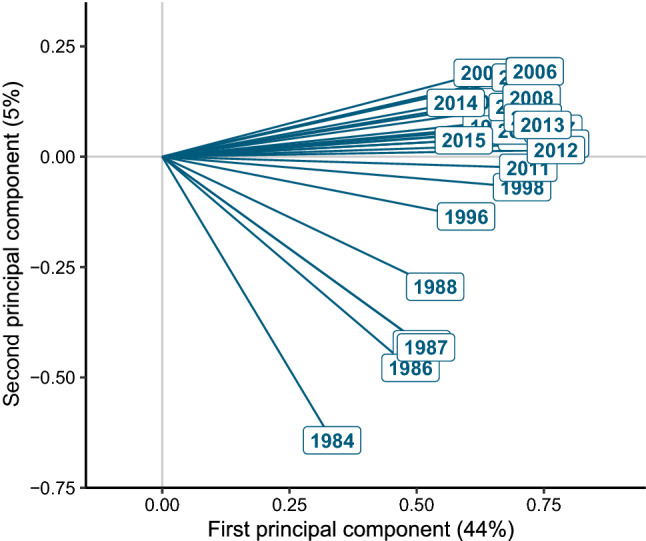
Representation of the years (1984–1988 and 1996–2015) sampled at the LTER-MC sampling site, in the Gulf of Naples, in the Interstructure map generated by the STATICO analysis. The map shows the relative importance of each year in the construction of a common species-environment space (the Compromise) and the similarity of the years in terms of biological-environmental dynamics. The length of the projection of each arrow on the first axis indicates the importance of each year in building the Compromise, while the angles among the arrows represent the correlations among the years, with same-direction arrows indicating similar years in terms of the species-environment structure. The years 1996–2015 were those highlighting the stable part of the evolution of the biological and environmental data over time, while 1984–1988 (1985 hidden by 1987) separated from the others, which indicates their lower contribution to the building of the Compromise space. The years 1989–1991 and 1995 were excluded from the analysis due to many missing data for environmental variables.

To investigate the seasonal and interannual fluctuations of the environmental variables in parallel with those of the phytoplankton community we used the STATICO analysis^37^, designed to describe the temporal evolution of the relationship between the phytoplankton and environmental variabilities in a common ordination space, a configuration named ‘Compromise’. According to the STATICO analysis, the relationship between phytoplankton communities and environmental variables remained relatively stable over the years at LTER-MC, as indicated by the high correlation among most of the years with the first axis (Fig. 4), which explained 44% of the total inertia. However, like in the Bray–Curtis autocorrelation analysis, the first years of the series (1984–1988) separated from the others, indicating a divergence in the species-environment relationship between those years and the rest of the time series (1996–2015), as also indicated by the lower median value of the loadings of the first years of the first PCA axis (0.15) compared to that of the other years (0.21). Accordingly, the data from the first part of the time series had a lower contribution to the building of the Compromise space with respect to the others, and were excluded from subsequent representations as trajectory maps, which would be scarcely reliable.

**Figure 5:**
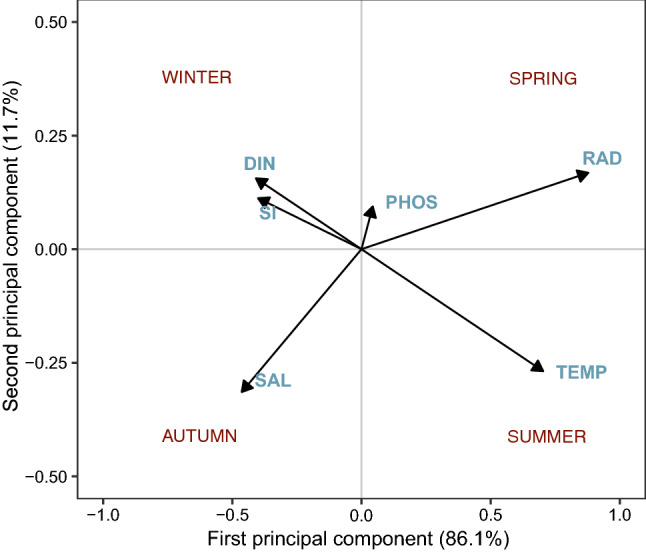
Species-environment space (Compromise) showing the relationships among environmental variables chosen for the STATICO analysis (SAL, salinity, TEMP, temperature; RAD, radiation; PHOS, phosphates; DIN, dissolved inorganic nitrogen, SI, silicates) relative to the period 1996–2015. Arrow lengths indicate the importance of each variable in defining the species-environment space, whereas the angles formed by the arrows indicate the correlation between the variables, with aligned arrows having a strong positive correlation, and those at right angles or opposite indicating no or negative correlations, respectively.

In the Compromise space (Fig. 5), representing the stable part of the biological and environmental relationship over time, the environmental gradient was mostly driven by radiation and temperature on the first axis (86.1% of the total inertia) and to a lower extent by salinity on the second axis (11.7%). The upper-left quadrant of the map represented the winter period, characterized by relatively high salinity, high nutrient concentrations and low temperature values. The spring and late-spring periods of the series covered the upper-right area of the map, with nutrients still relatively abundant but decreasing while approaching the bottom-right quadrant of the map, which marks the transition toward summer, characterised by the highest radiation and temperatures together with the lowest nutrient concentrations. The bottom-left quadrant of the Compromise map includes samples characterized by high salinity and relatively high nutrient concentrations; a condition typical of autumn at LTER-MC.

**Figure 6:**
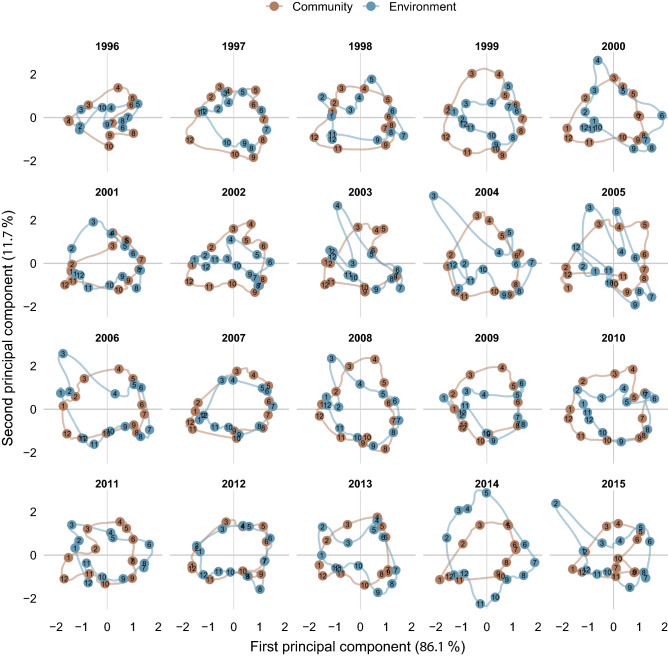
Yearly representation of phytoplankton community turnover compared to environmental variability. Monthly trajectories of phytoplankton community and environmental variabilities are projected on the common species-environment space (the Compromise) of Fig. 5. The labels on the trajectories indicate the months of the year. Bulges in the environmental trajectories in the upper quadrants in some years are related to nutrient pulses from terrestrial origin in winter or spring, while irregularities in the 3rd–4th quadrants are related to temperature anomalies in summer and autumn.

**Figure 7:**
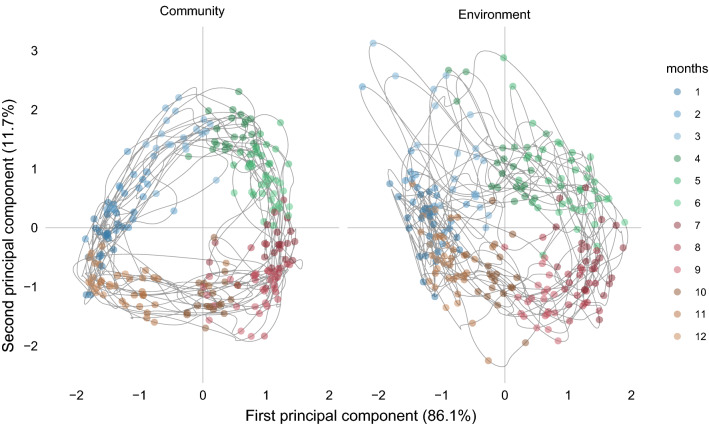
Temporal variability of phytoplankton community and environmental factors at LTER-MC relative to the period 1996–2015, all years superimposed. The monthly trajectories of both phytoplankton communities (left) and environmental variables (right) are projected on the Compromise space of Fig. 5. Filled, coloured circles refer to the months of the years. The comparison of the two plots highlights the relative regularity of the phytoplankton community turnover over the years, contrasted by the different shapes and irregularities of the environmental trajectories.

The trajectories in Fig. 6 represent the chronological projection of each month of a given year for the phytoplankton community and the environment, respectively, in the stable configuration of the species-environment relationship (the Compromise map, Fig. 5). Phytoplankton communities were characterized by quasi-circular trajectories relatively similar among the years, whereas environmental trajectories were less regular and often different from year to year. Particularly, in late winter and spring of 2003–2006, 2008 and 2014–2015, anomalous bulges related to nutrient pulses of terrestrial origin (low salinity) projected in the upper part of the plots, whereas anomalies related to high temperature were evident as irregular trajectories in the 3rd–4th quadrants in the summers and autumns of 2002–2005 and 2013–2015. In addition to these anomalies, environmental trajectories differed in both shape and breadth compared to phytoplankton ones (Fig. 7), again pointing at a quite variable environmental context experienced by the phytoplankton community from year to year. Interestingly, the community turnover pace was not homogeneous over the annual cycle but showed two phases of brisker transitions, seen as longer distances among monthly assemblages, namely from summer to autumn and from winter to spring, corresponding to the break and the formation of the thermocline, respectively.

### Identifying factors driving temporal phytoplankton variability

**Figure 8:**
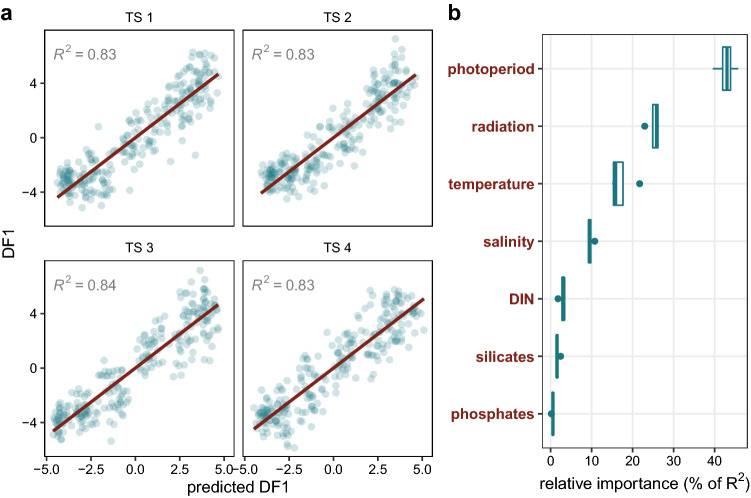
Relationship between phytoplankton community turnover and environmental parameters. (**a**) Multiple linear regression between the phytoplankton turnover indexed by the first discriminant function (DF1), extracted from the discriminant function analysis (DFA), and phytoplankton turnover predicted by environmental parameters. The regression analysis is performed on each of the 4 monthly time-series extracted from the whole weekly/biweekly time series. (**b**) Contribution of environmental parameters in predicting the temporal variability of phytoplankton community turnover, highlighting the prominent role of photoperiod in driving the phytoplankton community turnover.

To assess the relationship between environmental conditions and temporal phytoplankton variability, we estimated a monthly index summarising the temporal variability of the phytoplankton community using the sampling month as a predictor variable of phytoplankton species distribution and abundance in a discriminant function analysis (DFA). The DFA, performed on 4 distinct monthly time series (extracted from the complete 25-ys weekly/biweekly time series, see “Methods”) correctly predicted between 84 and 89% of the sampling months, with the first discriminant function (DF1) explaining between 36 and 42% of the overall predictive power, hence effectively summarising the temporal variability of the composition and abundance of the phytoplankton communities. The multiple linear regression between DF1 (as an index of temporal phytoplankton turnover) and environmental variables indicated phytoplankton communities as highly predictable based on environmental factors, as the models explained between 80 and 84% of the total temporal variability of their composition (Fig. 8a). In this analysis, a possible impact of correlated (collinear) variables was ruled out by VIF values lower than 6 in all the models. According to the Lindeman, Merenda and Gold's method for variance decomposition^38^, the photoperiod was by far the most important factor in explaining phytoplankton variability patterns (~ 42%) (Fig. 8b) followed by radiation and temperature (~ 26 and ~ 15% respectively), whereas salinity and inorganic nutrients seemed to play a marginal role, accounting for less than 10% of the total variability.

## Discussion

The analysis of the ca 32 ys-spanning time series gathered at the LTER-MC site confirms the dynamic nature of the inner Gulf of Naples, reflected in the high variability of the environmental conditions at both the seasonal and interannual scale. However, a remarkable regularity and stability are depicted by local phytoplankton communities, which may vary in total abundance but show a recurrent composition related to the periodical patterns of individual species and overall similar structure over the seasons from year to year.

High variability is evident in several environmental factors analysed over the whole time series. At the long term scale, the marked downward trends in all months and the cyclic interannual fluctuations of salinity, also reflected in the levels of the main nutrients, testify for the varying impact over the years of freshwater inputs from the coast. Apparently, the sampling area seems to be more frequently influenced by coastal inputs than by offshore waters in recent years, especially in spring, a pattern that is mostly driven by increased rainfall^39^. The temperature increase, particularly marked during the summer, is in line with the general warming trend reported for the whole Mediterranean Sea^40^. Besides trends, the environmental variability and its effects on phytoplankton abundance are also evident in the quite wide ranges shown by the climatology of physical, chemical and bulk biological variables considered in our study, namely, chlorophyll* a* and total cell number. These results are consistent with those from a previous meta-analysis investigating the temporal patterns of chlorophyll *a* from 84 coastal sites^9^, in which LTER-MC fell among the areas characterized by a marked seasonal dynamic but also strongly influenced by climatic shifts and processes of anthropic origin taking place at the interannual scale. This scenario translates into an environmental variability occurring at different temporal scales, in the form of isolated events, seasonal and cyclical fluctuations, and trends, which are fully evident in the present analysis of the multidecadal high-frequency sampling.

In contrast to the high environmental variability, our results reveal an impressive regularity in the annual occurrence of phytoplankton communities and individual taxa over the whole time series. The assemblages of the area, dominated by diatoms but including a high number of species belonging to all phytoplankton groups^41^, constitute a pool of biological entities encompassing a wide spectrum of differences in terms of phylogeny, sizes, shapes, life cycles and functional traits. In spite of such remarkable biodiversity, a clear periodicity is shared by more than 90% of the individual species investigated in this study. This regularity, reported in previous studies for a few species^35,42^, is fully substantiated in this study by quantitative and extensive analyses encompassing 81 species, all pointing at temporal regularity for the majority of them and for their assemblages. The consistency of seasonal patterns is also impressive for the shape of the annual phytoplankton cycle and the identity of the most representative taxa, which have remained relatively unchanged over the years: despite some variations, there is a usual increase from late autumn-early winter minima to late winter- early spring increase^13^, culminating with diatom-dominated late spring peaks which are followed by scattered and intense outbursts over the summer. These irregular summer peaks alternate with minima that reflect the occasional influence of offshore waters^43^, while a relatively regular, but lesser increase keeps on being recorded in autumn^35,44^.

Phytoplankton successional patterns in coastal temperate zones are argued to be determined by the chemical-physical environment, whereby nutrients, turbulence, temperature and radiance are the factors that have historically been associated with the control of microalgal growth (e.g.^23,45,46^). The seasonal interplay of these parameters would cyclically shape the environment creating predictable seasonal conditions and niches to which specific phytoplankton life forms and assemblages are suited^24,25,47^. In the case of the Gulf of Naples, the magnitude and frequency of nutrient supplies from the nearby coast may be rather variable from year to year, with unpredictable nutrient pulses shown both in the seasonal and interannual variations over the time series and in the results of the STATICO analysis. The lack of corresponding interannual variations in the phytoplankton community suggests a limited effect of nutrient pulses on phytoplankton successional dynamics even in cases when they were particularly intense, as in 2003–2006, 2008 and 2014–2015. This lack of response could depend on non-limiting nutrient levels in the area despite the seasonal signal often detected in their patterns. Indeed, in the decades 1984–2000, the water column was seldom depleted in nitrates and silicates even during the summer minima^35^, a condition that would also explain the frequent blooms of high nutrient-requiring and fast-growing diatoms even in this season^48^. As no significant negative trend in nitrates and silicates emerged from our analyses, the system appears to have maintained non-limiting nutrient conditions in recent years. Phosphates, which decreased in the last 10 years, have nevertheless maintained concentrations above limitation thresholds^35^, while the contiguity of oligotrophic water plays a role in dampening the effects of terrigenous nutrient inputs. Indeed, the intermittency of the different trophic regimes plays a role also in regulating phytoplankton successional dynamics, enhancing community diversity and preventing long-lasting dominance of a few species^43,48^. In these particular conditions, nutrients may occasionally become limiting, but not enough to restructure the phytoplankton community substantially, as it happens in other coastal areas^11,16,49^ and in particular in the northern Baltic Sea^50^. This does not imply that terrigenous nutrient inputs, from a broad perspective, are irrelevant in the study area, whereby their availability sets the general trophic conditions of the area. We would rather emphasize that the lack of constraint from persisting nutrient limitation enables the emergence of underlying species-specific biological processes for individual species, and, in a broader perspective, for the whole community.

As for other environmental parameters that are related to temporal changes in phytoplankton communities, the trajectories over the seasonal cycle reveal two phases of brisker changes, namely, in the transitions from winter to spring and summer to autumn, corresponding to faster shifts in the thermal exchange with the atmosphere and salinity that lead to the changes in the water column structure. Such regular switching states, also documented for the eukaryotic community in the oligotrophic Blanes Bay (Spain)^51^ and for bacterioplankton in the Beaufort Sea (North Carolina, USA)^52^ and Western English Channel^53^, highlight the relevance of the structuring and de-structuring phases of the water column, which may exert a prominent role in controlling the pace of the succession and changes in the community organization.

The relevance of environmental conditions is also highlighted by the peculiarity of the first years of the time series (1984–1991) with lower salinities, higher phosphate levels and higher biomass, already noticed in previous studies^13,35^. The changes already visible after 1988 are reflected in the lower similarity of the plankton communities to those of the following years (1996–2015) and also in the low contribution of the early years to the definition of a common biological and environmental space in the STATICO analysis. No clear explanations have so far been found for those changes, which could be related to the peculiar climatic dynamics that involved the entire northern hemisphere^54^ and were also reflected in the Mediterranean Sea at the end of the ‘80s. In those years, sudden changes in the atmospheric and hydrological systems had a significant impact on the biological systems of the Adriatic (eastern Mediterranean) and Ligurian (western Mediterranean) Seas, also affecting their planktonic systems^55^. It is plausible that such a widespread shift concerned our study area as well, but this hypothesis is hardly testable because sampling only started a few years before and was interrupted in the years that would correspond to the shift. It is remarkable though that the main components of the communities and their periodicity did not differ substantially not even between the first years of the series and the following years.

The temporal regularity of individual taxa together with the cyclic pattern of the entire community in the long term suggest that more important internal regulation processes may play a key role in the annual rhythms observed in our study. Several endogenous biological attributes such as life cycles, species-specific physiology and growth rates may translate into the temporally regular signal under the peculiar environmental setting of the Gulf the Naples. The prominent role of photoperiod, accounting for most of the temporal community variability in our analyses, indeed supports the importance of endogenous rhythms linked to the most recurrent variable in our otherwise variable coastal site. Signals of recurrent patterns like those recorded in this study have also been detected in other areas. The yearly bloom timing of diatoms species in Norwegian coastal waters hardly varied under environmental conditions changing from year to year, leading to the hypothesis that it was related to day-length-regulated germination of diatom resting spores^56^. That benthic stage germination could be responsible for the regular occurrence of plankton species was first proposed by Hansen^57^ based on his observation in the Kiel Bight and then confirmed by studies highlighting the role of endogenous regulation in germination timing of dinoflagellates cysts^58,59^. Yet a periodicity is also evident in species that do not form resting stages as the quite regular *Tripos* (= *Ceratium*) in the Kiel Bight^57^, or several taxa considered in the present study. Accordingly, whole-community analyses from different areas point at a lack of a clear relation between annual regularity and inorganic nutrients^60^ and show photoperiod explaining a large fraction of seasonal phytoplankton variability^61^. The records of regular seasonal patterns have probably been limited so far by the scarcity of time series including species-level phytoplankton data. As a matter of fact, recurrent seasonal patterns are being reported with increasing frequency in recent DNA-metabarcoding studies, which allow for a high taxonomic resolution for most taxa (e.g.^51,62,63^), with limited if any explanations by environmental variables other than photoperiod under variable environmental conditions, in terms of irregular inter-annual nutrient supply and salinity levels^64^. While most of the above-mentioned molecular studies focus on the smallest size-fraction or show recurrent patterns for a restricted part of the community, our study expands those results providing evidence for the recurrence of most taxa of the phytoplankton community over different size ranges which determines the overall seasonal regularity of the whole community.

In addition to photoperiod, other factors linked to the astronomic setting of our study site, notably temperature and irradiance, also partially explain the variability of phytoplankton community composition over the year. However, the prominent role of photoperiod suggests that this variable, rather than directly influence growth rates, acts as a signal for phytoplankton species to set their timing, thus getting them to grow in certain periods of the years that are most favourable to their ecological and reproductive success^65–67^. Indeed, phytoplankton communities and the large majority of individual species in the Gulf of Naples show diverse phenological patterns that cover the whole year, rather than being concentrated in periods of longer day length, which should be more favourable for all species and especially for diatoms^68^. The other environmental variables, i.e., temperature, salinity, nutrients, as well as biological ones (competition, grazing, parasites, etc.), would hence act at the evolutionary level as distal rather than proximal drivers, selecting populations that are set to grow in those periods. In this respect, the match of environmental conditions with functional traits^24,33,69^ should be seen as the result of an adaptive process rather than a cause-effect relationship. Like in terrestrial plants, phenological rhythms in these microbes could include a genetic component^70^ entrained by the signal provided by light and modulated by other environmental factors, which would explain a certain amount of phenological variability normally observed for phytoplankton.

While confirming the dynamic environmental conditions of the study area, our results have shown temporal recurrence for phytoplankton species and communities over almost three decades. These microbial species, rather than passively undergo environmental fluctuations, appear to have a quite marked degree of endogenous control that allows them to occupy regularly defined temporal niches and confer resistance to the short and long-scale environmental changes occurring in the area.

The stability and resistance in a variable environment under both anthropogenic and climatic pressures may have important implications for management, suggesting that phytoplankton community structure may not be a good indicator of environmental changes^71,72^. However, while this study has been focused on regular occurrence patterns and stability signals, possible ‘sentinels’ should probably be looked at in specific phytoplankton attributes, not considered in our analyses, such as diversity and size, or interannual variability and abundance trends of specific taxa. Detailed studies should address changes in the relative importance of smaller versus larger taxa, translating into trends for the average phytoplankton cell size^35^, changes in the frequency and intensity of winter blooms^13^ or variability in the abundance and phenology of potentially toxic species, considering the relevance of these aspects to ecosystem functioning and health, and their contribution to the environmental status assessment.

On the other hand, the lack of substantial changes in phytoplankton community structure over the studied period should not make us lower our guard on possible sudden evolution of the planktonic system. It should be kept in mind that the stability of plankton communities under continuous pressures over time may escalate into some abrupt change, as it has happened under regime shifts in other areas^12,54,73^. An example of such shifts, although of a relatively minor entity, is the unexplained change that occurred in the Gulf of Naples around the end of the ‘80s. This consideration reinforces the need to continue the existing time series and investigate them in more detail, to discern the temporal signals driven by biological internal components from the environmentally-driven ones, and to focus on minor changes and interannual variations that may be interpreted as early warning signals of changes.

## Methods

### Sampling area

The LTER-MC sampling site is located two nautical miles off Naples (40.81°N, 14.25°E), on a depth of ca 76 m (Supplementary Fig. S1), in an area subjected to strong anthropogenic pressure from one of the most populated areas of Europe. Land runoff supplies new nutrients to the surface layers, which are generally characterised by less salty waters (> 36) compared to the layers below (< 38.2)^74^. Nonetheless, because of the contiguity with the open Tyrrhenian Sea, the eutrophic state driven by land runoff inputs alternates with or overlaps to an oligotrophic state due to the intrusion of offshore waters. The boundaries and the extension of these two different subsystems depend mainly on physical factors and vary over the seasons^75,76^.

### Data

The LTER-MC phytoplankton dataset consists of abundance or presence data for ca 800 species obtained from more than 1150 Niskin bottle samples and an equal number of net samples collected in surface waters (0.5 m) fortnightly for the first 6 years and at weekly scale since 1995. Among them, abundance data are available for little more than 370 taxa, some of which consist of suprageneric groups (e.g., undetermined cryptophytes, flagellates, naked dinoflagellates and pennate diatoms). A 4-ys gap (1991 to early 1995) separates the first from the second part of the series, which in some cases have been analysed separately in this study, as specified below, due to the different time scales and marked differences between them in both environmental and biological variables^35^.

Physical and chemical data were obtained through in situ measurements or from samples collected simultaneously to phytoplankton sampling. Salinity and nutrient samples were routinely collected at 10 fixed depths (0, 2, 5, 10, 20, 30, 40, 50, 60 and 70 m), seven of which (0, 2, 5, 10, 20, 40 and 60 m) were also sampled for chlorophyll *a*. Temperature data were obtained at 10 depths with reversing thermometers in the years 1984–1991 and with continuous multiparametric profilers from 1995 onwards. Salinity was determined with a salinometer (Beckman mod. RS7C and subsequently Autosal Guildline Instruments) until 2002, thence temperature, salinity and pressure data were obtained by a CTD multiparametric profiler (Sea-Bird Electronics, 9–11 plus V2) were used to measure. Inorganic nutrient samples were collected from Niskin bottles into 20 mL polyethylene vials and immediately frozen. The concentrations of ammonia, nitrates, nitrites, phosphates and silicates were determined following Hansen and Grasshoff^77^. For chlorophyll *a* concentration, a variable volume of seawater was filtered under low vacuum and then extracted in 10 ml of neutralized acetone. All the data are routinely subject to quality check protocol, described along with detailed methods in Sabia et al.^78^. Radiation data were obtained from the NASA Langley Research Center (LaRC) POWER Project funded through the NASA Earth Science/Applied Science Program (https://power.larc.nasa.gov/).

Phytoplankton abundance data for ca. 370 taxa (mostly at species level) were obtained using an inverted light microscope at × 400 magnification, observing transects of a sedimentation chamber corresponding to an actual volume of 0.02–1.52 mL of seawater. The identification of individual taxa was subjected to intercalibration among years and different microscopists, some of which were active since the first years. Because the method provides sounder data for the most frequent and abundant taxa, a subset of these taxa was selected among taxonomically unambiguous entities, as close as possible to the species level, after removing aggregated groups of multiple unidentified taxonomic units. A threshold of 5% was used to include only taxa that had been virtually recorded at least twice per year. Yearly distributions were also checked visually, and 4 additional, well-identified taxa with a lower frequency (*Bacteriastrum furcatum*, *Skeletonema tropicum*, *Umbilicosphaera sibogae* and *Lioloma* sp.) were retained for the analyses for a total of 81 species investigated (Supplementary Table S1).

### Statistical analyses

The climatology of surface (average of 0, 2 and 5 m values) and deep (average of 10, 20, 30, 40, 50, 60 and 70 m values) physical (temperature and salinity, mixed layer depth), chemical (dissolved inorganic nitrogen-DIN, phosphates and silicates) and biological (chlorophyll *a*) variables was investigated by averaging data on the weekly scale. Monotonic upward or downward trends for the whole series and each month separately were analysed performing a Mann–Kendall test^79,80^ implemented in the R package ‘wq’^81^.

To characterize periodic properties and identify the dominant periods or frequencies, the time series of individual phytoplankton taxa’ abundance were submitted to the Lomb-Scargle periodogram analysis^82,83^. To test the null hypothesis that there was no periodic component in the data, the probability of getting random peaks equal to or higher than the peak in the original data was computed using 100 repeated randomisations of each original data sequence^84^ and setting the significance level at 0.01.

The temporal variability in the turnover of the selected taxa was investigated using the Bray–Curtis autocorrelation analysis^85,86^, in which we calculated the similarity of the community composition between pairs of samples collected at each possible time-lag in the time series. The time-lags were organized on a monthly scale, and the Bray–Curtis similarity was averaged among all the samples distant by time lags of 30 ± 4, 60 ± 4, 90 ± 4 days and so on till the 11,580 ± 4 days’ time-lag (almost 32 years), the latter consisting of 12 observations. Similarities averaged for each monthly time-lag were visualized on a bidimensional space as a function of the temporal distance among the samples (time lag), summarising both the seasonal and the interannual compositional variability of the phytoplankton community^85^. The autocorrelation analysis was performed separately on the entire time series (1984–2015) and on its second part (1996–2015) only, and for each analysis, we estimated the interannual variability performing a Mann–Kendall test^79,80^.

### Multivariate analyses

The relationship between interannual environmental fluctuations and variability in the phytoplankton community was investigated with the STATICO analysis^37^, an ordination technique designed to characterize the stable part in the dynamics of the relationship between the biological and environmental components of a system. Such a stable part, named ‘Compromise’, is used as a reference space on which the temporal evolution of the variability of the biological and environmental relationship is projected. Data were organized in yearly pairs of tables (biological and environmental data matrices) covering the period 1984–2015 excluding the years 1989–1995, either totally missing (1991–1994) or characterised by several gaps in the seasonal distribution of environmental variables. For each year, one table included monthly-averaged surface values of physical–chemical variables (temperature, radiation, salinity, dissolved inorganic nitrogen (DIN), phosphates and silicates), and the other monthly-averaged surface values of cell density of the 81 selected phytoplankton taxa, respectively. A co-inertia analysis was performed on each pair of tables producing a sequence of cross-covariance matrices analysed with a partial triadic analysis. Specifically, a set of RV coefficients (a measure between 0 and 1 describing the similarity between two matrices^87^) were computed for each couple of cross-covariance tables producing a matrix of correlations between the different years. Then, a PCA was performed on the RV coefficients’ matrix to quantify the similarity of the different tables (years) in a visual configuration named ‘Interstructure’. Finally, each yearly couple of tables was projected in the Compromise map in the form of trajectories. For details of the methods, see^36,37,88^.

The temporal variability of the phytoplankton community was further investigated using the discriminant function analysis (DFA), a multivariate technique that quantifies how well a certain combination of continuous variables discriminates between two or more levels of a categorical variable. The rationale behind the use of DFA is to extract a single multivariate index that describes the compositional variability of a community over time and to use that index as a response variable to environmental factors^53,89^. We performed the DFA using the time series of taxon abundances (the continuous variables) as predictors of the sampling month (the categorical variable), assuming that it would be possible to predict a certain temporal point along the time series based on the community composition at a given time. The DFA generates n-1 discriminant functions (with n = no of months), the first of which (DF1) provides the most overall discrimination among the groups (months), summarising the variability of the phytoplankton community in the LTER-MC time series. In order to increase the robustness of the results, we took advantage of the high sampling frequency of the 25 ys time series and extracted four different monthly time series from weekly/biweekly data, i.e., starting from ~ 52 observations for each year, we obtained four different time series composed of 12 annual observations.

The relationship between environmental factors and community variability was then tested performing a multiple linear regression using DF1 as a response variable and the environmental parameters (photoperiod, radiation, temperature, salinity, DIN, silicates, and phosphates) as independent variables for each of the 4 time-series generated. To assess the presence of any multicollinearity among the predictors, we estimated the variance inflation factor (VIF), which indicates collinearity for values higher than 10^90^. Then, we estimated the contribution of the predictors to the model’s total explained variance. One of the problems when decomposing the variance in regression models is that each order of the regressors generates a different decomposition of the sum of squares. Therefore, the contributions were calculated according to Lindeman, Merenda and Gold's method^38^. The LMG method is based on the sequential sums of squares of all regressors and takes into account the dependence of the order of the regressors in the decomposition process. Although computationally expensive, the method provides reliable results also when dealing with multicollinearity among predictors^91,92^.

## Supplementary Information

Supplementary Information.

## Data Availability

The datasets and the explained code used in the current study are organized in a private GitHub repository. The repository contents are available upon request to the corresponding authors through a shareable GitFront link.
