# Supplementary material for: Photoperiod-driven rhythms reveal multi-decadal stability of phytoplankton communities in a highly fluctuating coastal environment
Source: Sci Rep. 2022 Mar 10;12:3908. doi: 10.1038/s41598-022-07009-6 (PMC8913669; doi:10.1038/s41598-022-07009-6)
Supplement: Supplementary file 1 — Supplementary Information. [file 41598_2022_7009_MOESM1_ESM.pdf]

Photoperiod-driven rhythms reveal multi-decadal stability of phytoplankton communities in a highly fluctuating coastal environment

Lorenzo Longobardi[1*], Laurent Dubroca[2], Francesca Margiotta[1], Diana Sarno[3] & Adriana Zingone[1,3*]

[1]Integrative Marine Ecology Department, Stazione Zoologica Anton Dohrn, Villa Comunale, 80121 Naples, Italy.

[2]Institut Français de Recherche Pour l'Exploitation de la Mer, IFREMER, Laboratoire Ressources Halieutiques de Port-en-Bessin, 14520 Port-en-Bessin-Huppain, France

[3]Research Infrastructures for Marine Biological Resources Department, Stazione Zoologica Anton Dohrn, Villa Comunale, 80121 Naples, Italy

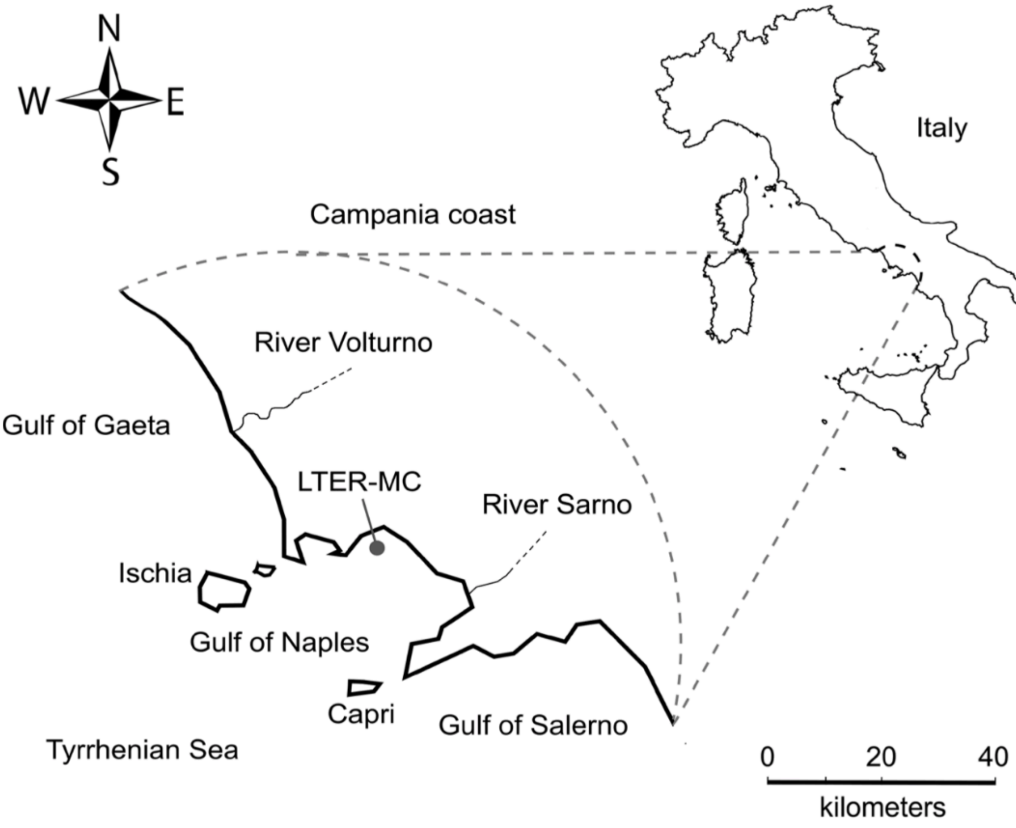

Figure S1. Map of the Gulf of Naples with the location of LTER-MC sampling station. Taken from Zingone et al. (2019).

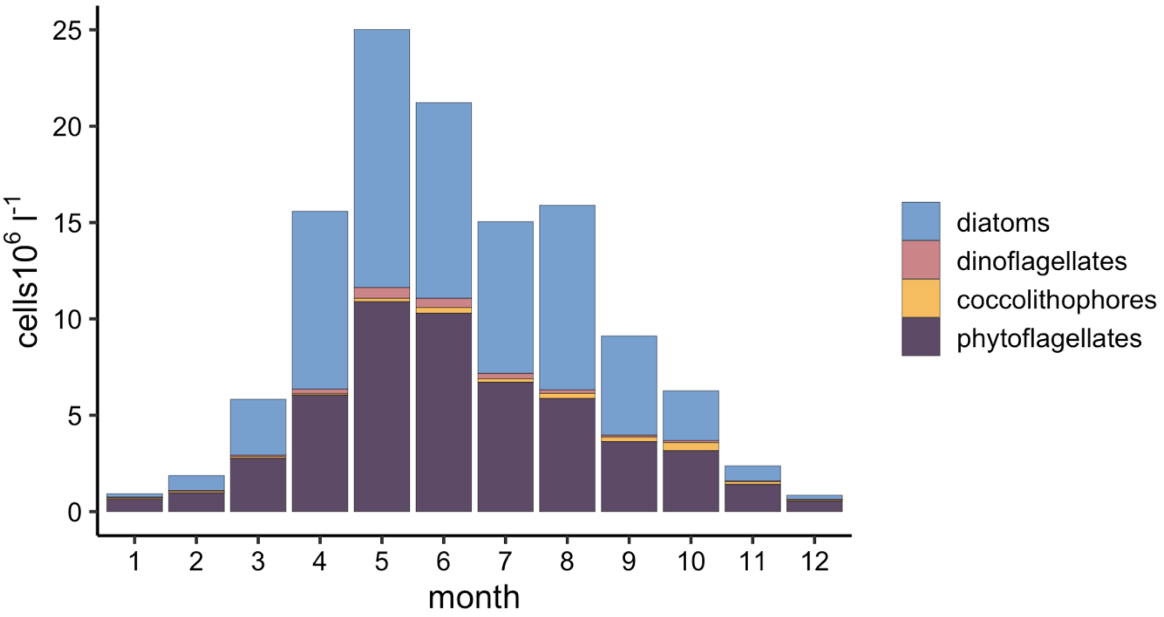

Figure S2. Monthly average abundance of surface phytoplankton functional groups (diatoms, phytoflagellates, dinoflagellates and coccolithophores) at LTER-MC during 1984-2015.

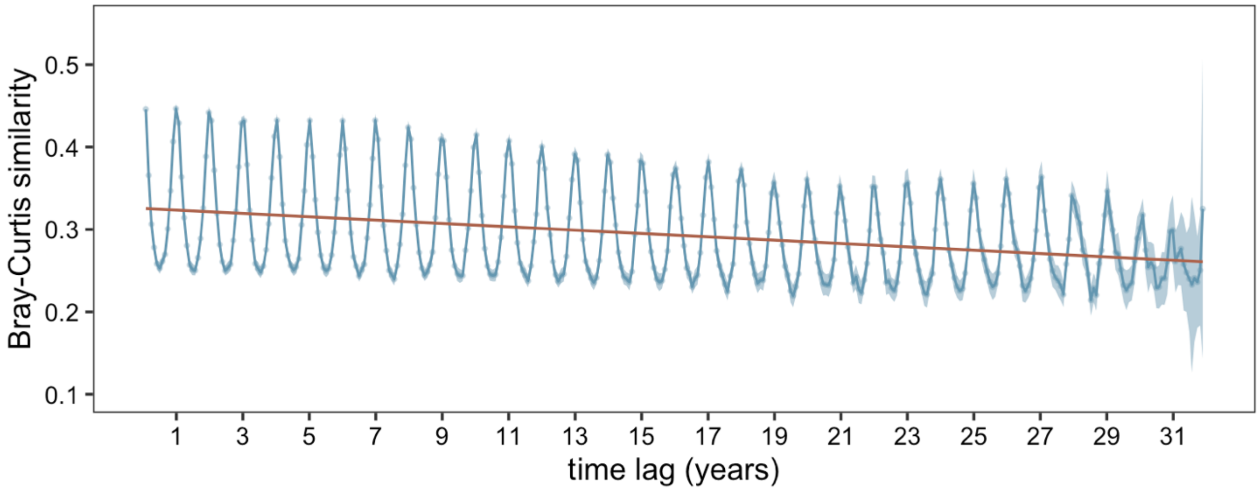

Figure S3. Interannual recurrence of phytoplankton communities shown by average. Bray–Curtis similarities (0: completely different communities; 1: identical communities; shaded area: 95% confidence interval) between all pairs of weekly samples separated by a given number of months of the series (time lag, x axis) during 1984–2015. The first point of the series represents the average and 95% confidence interval of the Bray–Curtis similarity values among communities sampled 30 ± 4 days apart in the time interval considered. Similarly, the second point represents the average Bray–Curtis similarity among all the communities sampled 60 ± 4 days apart, the point at 1 year is the average similarity among all the communities sampled at 360 ± 4 days apart, and so on. The decay of maximum and minimum average similarity values over time is probably influenced by the lower number of samples to compare for time lags of many years.

Table S1. Main statistics and results of periodicity' significance test of phytoplankton species during 1984-2015. The relative frequency and the average abundance are the proportion of the number of species' presences and the mean cell counts calculated over all samples respectively.

Taxa	Functional group	Phylum	Class	Lomb-Scargle' significance test	Relative frequency (%)	Average abundance (cells mL^{-1})	SD average abundance (cells mL^{-1})
Emiliania huxleyi	coccolithophores	Haptophyta	Prymnesiophyceae	periodic	81.11	99.841	444.918
Chaetoceros tenuissimus	diatoms	Ochrophyta	Bacillariophyceae	periodic	80.24	833.686	2667.684
Leptocylindrus danicus	diatoms	Ochrophyta	Bacillariophyceae	periodic	75.56	516.291	1459.909
Ollicola vangoorii	phytoflagellates	Ochrophyta	Chrysophyceae	periodic	74.09	49.375	108.720
Cylindrotheca closterium	diatoms	Ochrophyta	Bacillariophyceae	periodic	72.53	134.461	1061.541
Chaetoceros socialis	diatoms	Ochrophyta	Bacillariophyceae	periodic	58.23	361.161	1484.519
Paulinella ovalis	phytoflagellates	Cercozoa	Imbricatea	periodic	52.60	15.579	38.642
Skeletonema pseudocostatum	diatoms	Ochrophyta	Bacillariophyceae	periodic	51.91	724.356	3079.788
Pseudo-nitzschia delicatissima	diatoms	Ochrophyta	Bacillariophyceae	periodic	50.87	178.832	572.108
Pseudo-nitzschia galaxiae	diatoms	Ochrophyta	Bacillariophyceae	periodic	50.17	172.925	750.267
Leucocryptos marina	phytoflagellates	Cryptophyta	Cryptophyta incertae sedis	periodic	49.57	20.412	54.265
Pseudoscourfieldia marina	phytoflagellates	Chlorophyta	Pyramimonadophyceae	periodic	48.96	34.543	88.768
Cerataulina pelagica	diatoms	Ochrophyta	Bacillariophyceae	periodic	43.93	45.160	295.119
Skeletonema menzelii	diatoms	Ochrophyta	Bacillariophyceae	periodic	40.04	166.955	929.201
Pseudo-nitzschia pseudodelicatissima	diatoms	Ochrophyta	Bacillariophyceae	periodic	35.88	36.893	154.966
Bacteriastrum parallelum	diatoms	Ochrophyta	Bacillariophyceae	periodic	28.68	133.224	1193.967
Dactyliosolen fragilissimus	diatoms	Ochrophyta	Bacillariophyceae	periodic	28.25	12.162	52.529
Chaetoceros curvisetus	diatoms	Ochrophyta	Bacillariophyceae	periodic	27.56	25.021	92.875
Dinobryon faculiferum	phytoflagellates	Ochrophyta	Chrysophyceae	periodic	25.65	11.094	31.375
Pseudo-nitzschia galaxiae "small morphotype"	diatoms	Ochrophyta	Bacillariophyceae	periodic	25.30	11.971	51.865
Calciopappus caudatus	coccolithophores	Haptophyta	Prymnesiophyceae	periodic	25.22	9.122	30.542
Chaetoceros simplex	diatoms	Ochrophyta	Bacillariophyceae	periodic	22.53	35.496	166.768
Meringosphaera mediterranea	phytoflagellates	Ochrophyta	Xanthophyceae	periodic	22.36	2.729	10.984
Syracosphaera pulchra	coccolithophores	Haptophyta	Prymnesiophyceae	periodic	22.27	4.616	22.351
Thalassionema nitzschioides	diatoms	Ochrophyta	Bacillariophyceae	periodic	22.10	10.662	50.545
Chaetoceros "curvi-curvi"	diatoms	Ochrophyta	Bacillariophyceae	periodic	22.01	44.504	187.802
Minidiscus comicus	diatoms	Ochrophyta	Bacillariophyceae	periodic	20.54	87.400	831.800
Oxytoxum variabile	dinoflagellates	Myzozoa	Dinophyceae	periodic	18.63	1.532	6.508
Dactyliosolen blavyanus	diatoms	Ochrophyta	Bacillariophyceae	periodic	18.20	5.434	27.295
Pseudo-nitzschia multistriata	diatoms	Ochrophyta	Bacillariophyceae	periodic	17.24	9.069	44.712
Tenuicylindrus belgicus	diatoms	Ochrophyta	Bacillariophyceae	periodic	15.95	20.343	137.977
Thalassiosira rotula	diatoms	Ochrophyta	Bacillariophyceae	periodic	15.77	8.415	49.317
Syracosphaera molischii	coccolithophores	Haptophyta	Prymnesiophyceae	periodic	14.82	1.478	7.630
Prorocentrum triestinum	dinoflagellates	Myzozoa	Dinophyceae	periodic	14.56	7.427	40.292
Chaetoceros minimus	diatoms	Ochrophyta	Bacillariophyceae	nonperiodic	14.39	18.118	147.183
Calciosolenia brasiliensis	coccolithophores	Haptophyta	Prymnesiophyceae	nonperiodic	14.39	1.510	6.379
Ophiaster spp.	coccolithophores	Haptophyta	Prymnesiophyceae	periodic	14.04	0.555	2.595
Acanthoica quattrospina	coccolithophores	Haptophyta	Prymnesiophyceae	periodic	13.43	0.703	3.509
Dictyocha fibula	phytoflagellates	Ochrophyta	Dictyochophyceae	periodic	12.57	0.330	1.508
Asterionellopsis glacialis	diatoms	Ochrophyta	Bacillariophyceae	periodic	12.48	9.622	51.439
Rhabdosphaera clavigera	coccolithophores	Haptophyta	Prymnesiophyceae	nonperiodic	12.22	1.016	4.160
Thalassionema bacillare/frauenfeldii	diatoms	Ochrophyta	Bacillariophyceae	periodic	12.13	2.142	10.770
Lessardia elongata	dinoflagellates	Myzozoa	Dinophyceae	periodic	11.87	2.251	9.883
Eutreptiella spp.	phytoflagellates	Euglenozoa	Euglenoidea	periodic	11.79	9.968	230.784
Chaetoceros affinis	diatoms	Ochrophyta	Bacillariophyceae	periodic	11.79	7.621	39.183
Proboscia alata	diatoms	Ochrophyta	Bacillariophyceae	periodic	11.79	1.716	8.360
Dactyliosolen phuketensis	diatoms	Ochrophyta	Bacillariophyceae	periodic	11.44	2.740	20.593
Guinardia striata	diatoms	Ochrophyta	Bacillariophyceae	periodic	10.66	2.199	13.753
Apedinella radians	phytoflagellates	Ochrophyta	Dictyochophyceae	periodic	10.23	1.725	9.830
Thalassiosira mediterranea	diatoms	Ochrophyta	Bacillariophyceae	periodic	9.62	12.108	144.521
Chaetoceros diadema	diatoms	Ochrophyta	Bacillariophyceae	periodic	9.10	11.441	68.375
Chaetoceros throndsenii	diatoms	Ochrophyta	Bacillariophyceae	periodic	8.93	38.160	378.091
Chaetoceros contortus	diatoms	Ochrophyta	Bacillariophyceae	periodic	8.93	17.016	102.922
Lauderia annulata	diatoms	Ochrophyta	Bacillariophyceae	periodic	8.93	2.193	11.314
Heterocapsa niei	dinoflagellates	Myzozoa	Dinophyceae	periodic	8.67	2.746	14.841
Chaetoceros protuberans	diatoms	Ochrophyta	Bacillariophyceae	periodic	8.06	3.544	21.745
Diplostauron cf. elegans	phytoflagellates	Chlorophyta	Chlorophyceae	periodic	7.89	3.082	19.276
Solenicola setigera	phytoflagellates	unassigned	unassigned	periodic	6.85	4.202	36.203
Chaetoceros peruvianus	diatoms	Ochrophyta	Bacillariophyceae	nonperiodic	6.85	0.480	3.168
Leptocylindrus mediterraneus	diatoms	Ochrophyta	Bacillariophyceae	periodic	6.67	0.496	3.802
Chaetoceros costatus	diatoms	Ochrophyta	Bacillariophyceae	periodic	6.59	4.551	27.828
Chaetoceros pseudocurvisetus	diatoms	Ochrophyta	Bacillariophyceae	periodic	6.41	3.851	29.346
Chaetoceros decipiens	diatoms	Ochrophyta	Bacillariophyceae	periodic	6.41	1.711	13.331
Leptocylindrus convexus	diatoms	Ochrophyta	Bacillariophyceae	periodic	6.33	8.755	63.990
Lithodesmium cf. variabile	diatoms	Ochrophyta	Bacillariophyceae	periodic	6.33	2.395	23.235
Dinobryon coalescens	phytoflagellates	Ochrophyta	Chrysophyceae	periodic	6.15	7.670	71.109
Chaetoceros anastomosans	diatoms	Ochrophyta	Bacillariophyceae	nonperiodic	6.15	3.651	31.924
Algirosphaera robusta	coccolithophores	Haptophyta	Prymnesiophyceae	periodic	6.15	0.150	1.247
Coronosphaera mediterranea	coccolithophores	Haptophyta	Prymnesiophyceae	periodic	5.98	0.118	0.803
Protoperidinium bipes	dinoflagellates	Myzozoa	Dinophyceae	periodic	5.89	0.744	4.436
Chaetoceros wighamii	diatoms	Ochrophyta	Bacillariophyceae	periodic	5.46	17.945	163.828
Minutocellus polymorphus	diatoms	Ochrophyta	Bacillariophyceae	periodic	5.37	31.354	476.638
Pseudo-nitzschia fraudulenta/subfraudulenta	diatoms	Ochrophyta	Bacillariophyceae	periodic	5.29	1.945	14.986
Chaetoceros single sp.1	diatoms	Ochrophyta	Bacillariophyceae	periodic	5.11	82.167	1139.217
Chaetoceros diversus	diatoms	Ochrophyta	Bacillariophyceae	periodic	5.11	10.012	161.038
Eucampia zodiacus f. cylindricornis	diatoms	Ochrophyta	Bacillariophyceae	nonperiodic	5.11	0.858	6.935
Sphaerocalyptra quadridentata	coccolithophores	Haptophyta	Prymnesiophyceae	periodic	5.03	0.985	7.016
Bacteriastrum furcatum	diatoms	Ochrophyta	Bacillariophyceae	periodic	4.68	2.951	49.851
Skeletonema tropicum	diatoms	Ochrophyta	Bacillariophyceae	periodic	4.51	3.840	28.527
Lioloma sp.	diatoms	Ochrophyta	Bacillariophyceae	periodic	4.51	0.500	3.661
Umbilicosphaera sibogae	coccolithophores	Haptophyta	Prymnesiophyceae	periodic	3.21	0.050	0.365
